# Computer tomographic investigation of subcutaneous adipose tissue as an indicator of body composition

**DOI:** 10.1186/1751-0147-51-28

**Published:** 2009-07-01

**Authors:** Fintan J McEvoy, Mads T Madsen, Mai B Nielsen, Eiliv L Svalastoga

**Affiliations:** 1 Department of Small Animal Clinical Sciences, Faculty of Life Sciences, University of Copenhagen, 32 Dyrlaegevej, DK-1870 Frederiksberg C, Denmark; 2 Danish Pig Production, 3 Axeltorv, DK-1609 Copenhagen, Denmark

## Abstract

**Background:**

Modern computer tomography (CT) equipment can be used to acquire whole-body data from large animals such as pigs in minutes or less. In some circumstances, computer assisted analysis of the resulting image data can identify and measure anatomical features. The thickness of subcutaneous adipose tissue at a specific site measured by ultrasound, is used in the pig industry to assess adiposity and inform management decisions that have an impact on reproduction, food conversion performance and sow longevity. The measurement site, called "P2", is used throughout the industry. We propose that CT can be used to measure subcutaneous adipose tissue thickness and identify novel measurement sites that can be used as predictors of general adiposity.

**Methods:**

Growing pigs (*N *= 12), were each CT scanned on three occasions. From these data the total volume of adipose tissue was determined and expressed as a proportion of total volume (fat-index). A computer algorithm was used to determined 10,201 subcutaneous adipose thickness measurements in each pig for each scan. From these data, sites were selected where correlation with fat-index was optimal.

**Results:**

Image analysis correctly identified the limits of the relevant tissues and automated measurements were successfully generated. Two sites on the animal were identified where there was optimal correlation with fat-index. The first of these was located 4 intercostal spaces cranial to the caudal extremity of the last rib, the other, a further 5 intercostal spaces cranially.

**Conclusion:**

The approach to image analysis reported permits the creation of various maps showing adipose thickness or correlation of thickness with other variables by location on the surface of the pig. The method identified novel adipose thickness measurement positions that are superior (as predictors of adiposity) to the site which is in current use. A similar approach could be used in other situations to quantify potential links between subcutaneous adiposity and disease or production traits.

## Background

Subcutaneous adipose tissue (SAT) changes in its dimensions and properties during growth and according to diet. It forms a continuous layer of tissue covering the body and may be thought of as a "fat mantle". Simple thickness measurements of this tissue layer can be used to describe body composition and are important in production animals when food conversion efficiency has to be maintained, for predicting and targeting meat quality and for aspects of husbandry including reproductive performance and longevity [1-4]. Historically in pigs, these thickness measurements are made at a particular site designated "P2". This is situated 6.5 – 7 cm from the mid dorsum at the level of the last rib [5]. In recent years, this dimension has been measured using images from B-Mode ultrasound imaging equipment or from A- mode ultrasound units, which yield numerical data only. The choice of the measurement site is partly historical; prior to the use of ultrasound these sites were evaluated by palpation [6].

Computer tomography (CT) images display clearly the partition between adipose tissue which has CT numbers less than zero and its bounding tissues (skin and muscle) which have CT numbers greater than zero [7]. Images are free from magnification so that measurements of thickness can be made directly from the image. Modern helical CT machines are capable of scanning large regions in short times. A whole body scan in a pig may take 80 seconds or less. In that time the entire body may be sampled in continuous slices of equal thickness. Thickness measurements can be made from both CT and ultrasound images. CT is not practical for use under farm conditions but with appropriate analysis techniques, we propose that the vast quantity of image data contained in CT images can be used in preliminary investigations to measure an almost infinite number of sample points which in turn can be tested for usefulness. This may allow one to identify a single or a small number of points that correlate with a desired outcome, (body composition in this paper) that in future can be measured by ultrasonography, which by contrast, is readily applied under farm conditions.

This paper describes the use of CT to map the thickness of the fat mantle in growing pigs and tests multiple sampling points in order to identify optimal sites for the ultrasound assessment of adiposity. It examines the hypothesis that a systematic approach to the selection of measuring sites is possible using computer aided analysis of CT images. This will allow identification of potential measurement sites to predict adiposity that are superior to those that are currently used in this species.

## Methods

### Animals

Twelve Danish Landrace female pigs were used. At the outset of the study, their mean age (standard deviation in parenthesis) was 116 (1.1) days, and mean body-mass was 51.7 (4.0) Kg. They were fed a standard diet for use in gilts, with energy content ranging from 1 to 1.2 FEso/Kg. The study was approved by the Danish National Animal Ethics Council.

### Computer tomography scanning

CT images were collected in 3 scanning sessions, the first at the start of the experiment and the others, 38 and 64 days later. For each scanning, the pigs were sedated using Azaperone (0.1 to 0.2 mg/kg, i.m.) to prevent movement blur on the images. All scans were performed with the pigs in sternal recumbency. A single slice helical computer tomography (CT) scanner (Emotion, Siemens, Germany) was used. Imaging parameters were set as follows: 5 mm slice thickness, 1.3 pitch, 110 KV and 137 mAs. Scan time using this protocol was approximately 80 seconds per pig allowing collection of transverse images of the entire body region, from the level of the shoulders caudally to the level of the hips. For analyses a reconstruction algorithm favoring contrast resolution was used.

### Ultrasound scanning and measurements

The thickness of skin plus SAT was determined at the "P2" site using ultrasound (Siemens, Acuson Sequoia fitted with a 14 MHz transducer). The site used was found by palpation; it is situated 7 cm lateral to the dorsal spinous process at the level of the most caudal point of the last rib. The combined thickness of skin and SAT was measured using the scanner's software. These measurements were thus the standard "P2 measurements" used in the pig industry.

### Image analysis

The CT unit generates an image stack composed of multiple consecutive transverse images or slices. In order to allow image data from one individual to be combined with that from another, it was necessary to reformat the image stacks so that a particular slice location from one individual matched the location at the same slice number in another individual. This was done by combining data from adjacent slices so that the data was reduced to exactly 101 "pseudo-slices" in all individuals and setting reference anatomical landmarks at particular slice numbers. For each pig the slice at the level of the most caudal lumbar vertebra and at 6, 11 and 16 spinous processes cranial to that were numbered 1, 39, 73 and 101 respectively. Slices for the body regions between these reference points were equally spaced and numbered consecutively. Typically the level of the last rib was located at the level of pseudo-slice 39. This was achieved using a dedicated computer program written using commercial programming software (Matlab 7.0, MathWorks Inc, Cambridge MA, USA).

### Subcutaneous adipose tissue thickness

The same software (Matlab) was also used to determine the thickness of skin plus SAT in each image slice in a semi-automated fashion. Briefly this involved determination of the length of the outer perimeter of the side being examined; this distance was divided into 100 equal parts by means of 101 measure points. The combined skin plus SAT thickness at each of these 101 points was measured by the dedicated algorithm. This resulted in 101*x*101 measurements per pig per scan. Right and left was assumed to be identical so only one complete half, chosen randomly, from mid dorsum to the ventral midline on each image was analyzed.

### Body composition estimation

In addition to determinations of skin plus SAT thickness described above, an estimate of general adiposity was also made from the CT data. This was done for each pig and each scan. For each scan, the number of voxels in the entire data set with CT numbers between -300 and +300 Hounsfield units (HU) was plotted. This curve showed individual peaks, one for adipose tissue and another for non-adipose soft tissues. By examining the areas under the curve for adipose tissue, the total adipose tissue volume was determined. A detailed account of the method used to generate these curves and of the determination of reference CT number ranges and volumes has been reported previously [8]. The whole volume of the pig for the region scanned was also determined. The ratio of the volume of adipose tissue to total volume expressed as a percentage (fat-index) was calculated, taken as an indicator of general adiposity and used in the statistical analyses. It should be noted that this is an estimate of body-fat by volume and not by mass.

### Correlation between sub-cutaneous adipose tissue thickness and general adiposity

Data from each measurement point for each scan session was entered into commercial statistical software (SAS, version 8.2, SAS Institute, Cary, NC, USA). The number of measurement points for later analysis was reduced as follows. The average Pearson correlation coefficient, indicating the association between fat-index and the combined skin plus SAT thickness at each measure point was calculated. The sum and range of these values for the three scans at each measure point was displayed in tabular form. A search was made for measure points where both the sum of correlation coefficients was maximal and their range was minimal. This allowed the selection of sites that had an overall high correlation with fat-index and low variation between scan sessions. Four such sites, representing different regions of the body surface, were selected as candidate measurement points to test in a mixed linear model. The model was run using the "Mixed Procedure" in SAS. The continuous variables, body-mass and thickness at the 4 candidate sites and the categorical variable "session" were included in the model as predictors of fat-index. A stepwise removal of explanatory variables from the model was used to identify the most important.

## Results

**Figure 1:**
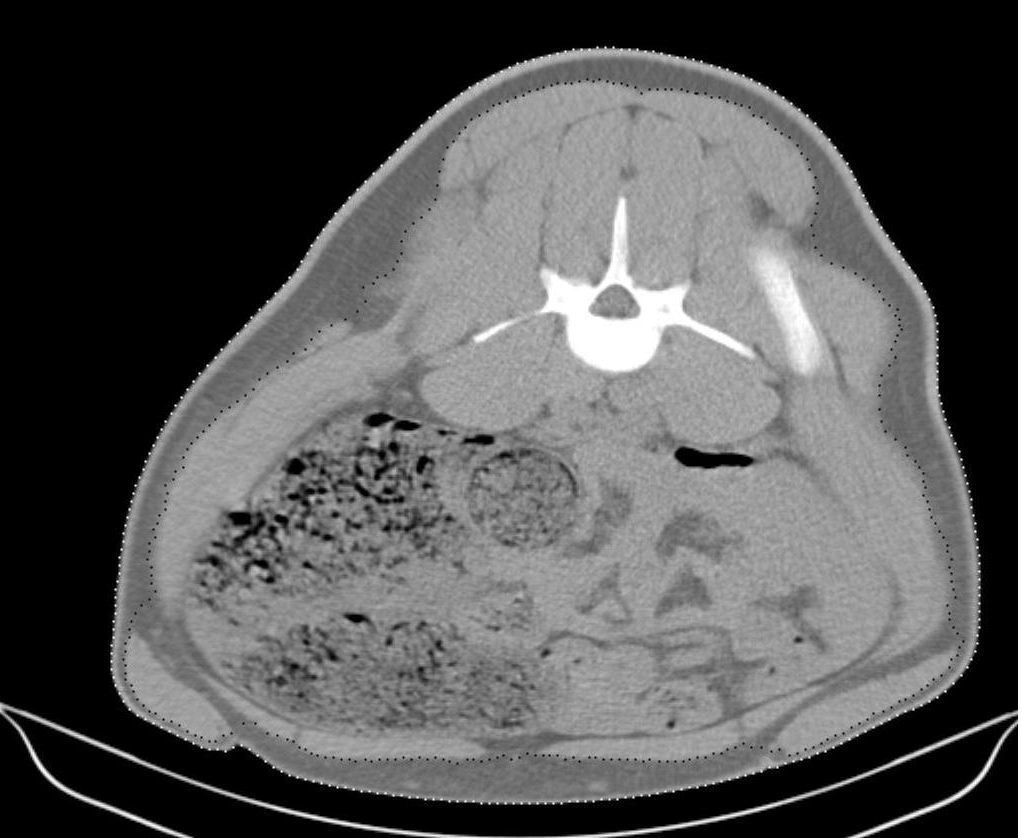
**Computer tomography image**. Transverse CT image showing the automatically determined boundaries. The outer limits of the skin are marked with white dots; the inner margin of subcutaneous adipose tissue is marked with black dots. Note in this particular image the algorithm misrepresents the desired boundaries in places because it was interrupted by discontinuities within the adipose tissue.

All pigs increased in body-mass between scans. The mean body-mass and its standard deviation (in parenthesis) were 94.9 (6.5) and 111.6 (13.1) Kg at the second and third scan sessions respectively. An image showing the points identified for measurement is shown in Figure 1. It can be seen that the algorithm accurately identifies the outer aspect of the skin and at most sites the innermost surface of the subcutaneous adipose tissue. The individual measurements thus made, correspond to skin thickness plus subcutaneous adipose tissue thickness. In places (for example ventrolaterally in Figure 1) the algorithm misrepresented the full thickness of the layer due to the presence of soft tissues (segments of the Cutaneus trunci muscle) within (as opposed to being deep to) the subcutaneous adipose tissue. On visual inspection, these images were identified, deleted and replaced by neighboring slices. These misrepresentations were thus not considered to have any significance on the overall results.

**Figure 2:**
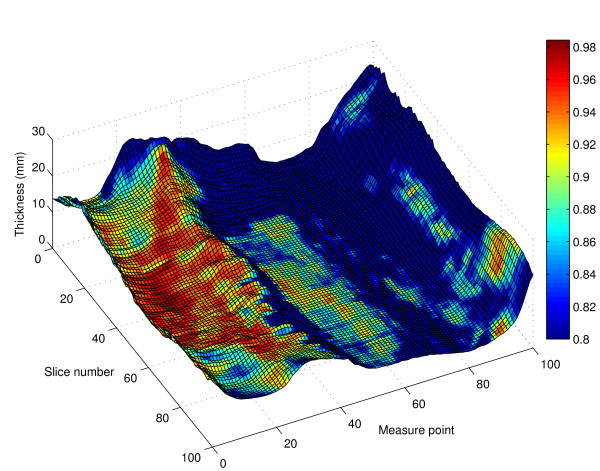
**Average subcutaneous adipose tissue thickness**. Wireframe plot of the average subcutaneous adipose tissue thickness (*N *= 12) at the final scan session, over one side of the body surface. The x-axis, labeled "Measure point", shows position along the circumference of the pig (zero is dorsal and 101 is ventral). The z-axis, labeled "Slice number" describes distance along the length of the pig (zero is caudal, 101 is cranial). The y-axis, labeled "Thickness (mm)" shows the depth of skin plus subcutaneous adipose tissue in mm. The colour drape indicates correlation between skin plus subcutaneous adipose tissue thickness and total subcutaneous adipose tissue volume. Correlation coefficient values < 0.8 have been truncated to 0.8 for this figure, so that the full colour scale can be applied to values between 0.8 and 1.

**Figure 3:**
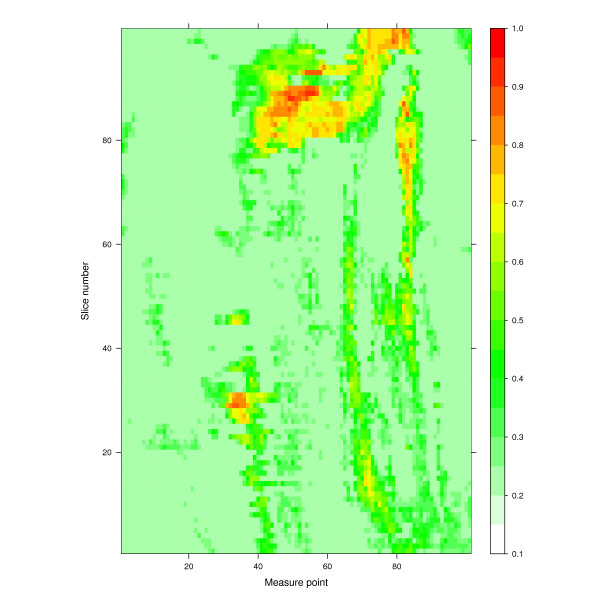
**Correlation between combined skin and subcutaneous adipose tissue thickness and general adiposity**. Level-plot showing the correlation at the last scan (*N *= 12) between the combined skin and subcutaneous adipose tissue thickness and general adiposity (fat-index), as defined in the text. The x-axis, labelled "Measure point", shows position along the circumference of the pig (zero is dorsal and 101 is ventral). The y-axis, labelled "Slice number" describes distance along the length of the pig (zero is caudal, 101 is cranial). The colour scale indicates the correlation coefficient between skin plus subcutaneous adipose tissue thickness and general adiposity (fat-index). Correlation coefficient values < 0.2 have been truncated to 0.2 for this figure so that the full colour scale can be applied to values between 0.2 and 1.

The average thickness measurements from all pigs at the final scan session and the average correlation between thickness at each measure point and total mantle volume for the final scan session is illustrated in Figure 2. This figure demonstrates the strength of the currently used P2 site, which is situated at slice 39 in a region of high correlation with fat mantle volume. Figure 3 shows the average of the correlation coefficient for the association between skin plus SAT thickness and fat-index, at each measure point at the final scan session.

The statistical model identified age (as represented by session number) and two measurement points, which were the best predictors of fat-index. These measure points were on slice 64 at measurement point 36 (designated "M") and on slice 93 at measure point 83 (designated "N"). In terms of surface anatomy, if a transverse slice through the animal is considered a clock-face with the 12 o'clock position at the dorsal mid-line and the 6 o'clock at the ventral mid-line, then measure point "M" is found 4 intercostal spaces cranial to the caudal extremity of the last rib at the 2 or 10 o'clock position and measure point N is found 5 intercostal spaces cranial to the "M" measure point at the 5 or 7 o'clock position. If *T*_*M *_and *T*_*N*_, are the thicknesses (in mm) of adipose tissue at positions "M" and "N" respectively, then:



where:

A is a variable according to age as follows;

-0.03914 for zero to 116 days

-0.02628 for 117 to 154 days

and

zero for 155 to 182 days respectively.

Over these age ranges, this relationship gives a correlation coefficient of 0.87 (*P *< 0.001) for fat-index. This is a superior model for predicting fat-index, to the use of ultrasound measurements at the P2 site alone, where the association between tissue depth and fat-index had a correlation coefficient of 0.10 (*P *= 0.38). Of the two measure points selected for the model, point "N" contributes most to the model.

## Discussion

CT has been used previously in pigs and other production animals, in companion animals and in humans to determine body composition [9-12]. The whole-body radiation dose for each pig is estimated at 15 mSv based on recent literature [13]. This is not an insignificant dose but is below current annual whole body dose limits for humans working with ionizing radiations. Since animals were anesthetized no dose to personnel was associated with the procedure. For this study a ratio, the fat-index was taken as an indicator of adiposity; the statistical analysis was directed at producing a model that best predicts this ratio. The method requires that a CT number range be defined for adipose tissue. Various approaches can be taken for this. At its most basic, one can simply define all tissue less than zero but greater than -300 HU as adipose, or alternatively one can make an estimate based on the frequency distribution of the voxel CT number for each pig for each scan as was done in this study. The criterion used for defining adipose tissue will influence the volume estimate for this tissue on CT; this issue has been addressed in a previous study [8]. Fat-index estimation also requires an estimate of body volume. This quantity will depend to some extent on the degree of abdominal distention due to bowel contents. Thus the estimates of both the nominator and denominator used in determination of fat-index are subject to experimental variation. Fat-index however was considered a useful guide to general adiposity as it includes both visceral and subcutaneous adipose tissue and its use has been validated for this purpose in rabbits [14].

Automation in the image analysis was critical to this study. CT images are essentially digital maps of the linear attenuation coefficient for x-rays, displayed voxel by voxel. There are many examples in the literature of different automation strategies using CT images [15,16] and also in other cross sectional imaging modalities [17]. The algorithm used in this study was written to produce measurements that mimic those obtainable by ultrasound. CT images do not permit differentiation of the various layers of adipose tissue, typically 2 or 3 in number at the P2 site, which may be seen on gross specimens and on ultrasound images. This may be of importance since it is known that the rate of change in thickness and likely the physiological function differs between layers [18,19]. Notwithstanding this, ultrasound imaging is more commonly used to determine the total thickness of adipose tissue and the algorithm was successful in that measurement. In all it generated over 300,000 thickness measurements from the 3 scan sessions. The number illustrates the wealth of information contained in CT images. Each measurement could theoretically have been collected using ultrasound, but it is clear that such a task would be physically impossible to accomplish.

The CT data-set permits slice reconstruction. Thus while the absolute number of slices obtained from pig to pig and from scan to scan differed as a function of the animal's length, slice reconstruction allowed the generation of 101 "pseudo-slices" for every scan. These were further registered to fixed anatomical landmarks so that data from any particular CT slice could be merged with that from the corresponding slice in the other pigs. This permitted statistical analysis of the image data. The quantitative aspects of CT imaging modality are being increasingly recognized in many areas including in the study of obesity [20,21]. Figures 2 and 3 illustrate the extent of the data. They display point by point, the average thickness and the correlation between thickness and total SAT volume (Figure 2), and between thickness and fat-index (Figure 3). It is interesting to note in Figure 2 that the traditional P2 site is situated in a large zone of optimal correlation with total SAT volume.

The model derived in this study includes a factor for age. In the growing pigs examined here, body-mass and size will alter with age. It is likely that if a model is required for animals that are not growing, another factor to reflect body size, for example, body-mass or body circumference would be required in the final model. Age and two measurement sites were identified as important predictors of total adiposity in these growing pigs. The measurement sites have been determined on the basis of their ability to predict total adiposity. The use of CT permitted a total coverage of potential measurement points. All were considered accessible for measurement in this paper. Some sites may however, be too difficult to measure under field conditions. Such sites can be rejected in the analysis and others identified from those remaining.

If important health and production traits are influenced by the degree of general adiposity then the CT analysis described in this paper has identified novel sites that may be directly useful, and possibly more useful than the current P2 site. This however should be tested in experiments where repeated thickness measurements are made from these sites and where data for each trait of interest e.g. reproductive performance, sow longevity or meat characteristics, are available. While this study uses a specific question posed by the pig industry to illustrate the contribution CT analysis, an understanding of the correlation between simple non-invasive measurements and general adiposity is important in many veterinary species and in humans, were clinical obesity is of major concern. The experimental approach described here, can be readily applied to other investigations concerned with adiposity.

A rapidly developing area in medical imaging concerns parametric mapping. Statistical parametric mapping uses images whose pixel values are statistics [22]. This approach to image interpretation provides a means by which complex statistical trends can be appreciated by the viewer. The plots in Figures 2 and 3 can be considered parametric maps since they display how correlation varies with location. They offer a new ways of viewing function and probabilities.

## Conclusion

We have identified novel sites that are indicators of general adiposity (fat-index) and are of interest to the pig industry. Topographical analysis shows that the current "P2" site is situated in an area of low local variation and optimal correlation with the total volume of SAT. The investigative approach used in this paper provides a complete data set of all possible sub-cutaneous adipose tissue thickness measurements and can be used to investigate potential sites of optimal correlation with various adipose tissue related traits.

## Competing interests

The authors declare that they have no competing interests.

## Authors' contributions

FM conceived and participated in the design of the study, carried out the ultrasound and CT examinations and drafted the paper. MTM participated in the design of the study and provided feeding and management protocols for the animals used. MBN performed the statistical analysis. ES participated in the design of the study. All authors read and approved the final manuscript.
